# T103. ODIP (OUTIL DE DIAGNOSTIC INFORMATISé DES PSYCHOSES / PSYCHOSIS COMPUTERIZED DIAGNOSTIC TOOL): A NEW, SIMPLE METHOD FOR GENERATING DSM DIAGNOSES FOR PSYCHOTIC DISORDERS

**DOI:** 10.1093/schbul/sby016.379

**Published:** 2018-04-01

**Authors:** Jean-Romain Richard, Baptiste Pignon, Franck Schurhoff, Andrei Szoke

**Affiliations:** INSERM

## Abstract

**Background:**

OPCRIT was designed as a powerful tool to diagnose psychotic and affective psychoses. It has been frequently used in international psychiatric research. However, with 90 items it is time-consuming to complete and the diagnoses provided include many which are no longer used. Furthermore, this application is no updated for certain operating systems or psychiatric classifications.

For these reasons, we have developed, a similar but much simpler tool focused on DSM classification of affective and non-affective psychoses.

**Methods:**

ODIP is based on the DSM-IV psychotic disorders classification, focusing on psychotic disorders (affective and non-affective). We identified 13 criteria that allow for the distinction between affective disorders with psychotic features (Bipolar or Depressive episode), schizophrenia, schizophreniform, schizoaffective, delusional, brief or non-specified psychotic disorders. We also designed a form to collect data on these 13 items.

To assess how ODIP performs we tested it against the more complete OPCRIT and discordances in diagnosis were compared with the clinical diagnosis or, in a subsample of patients, with a research diagnosis.

This was done in a total sample of 464 patients with a first episode of psychosis.

First, we observed that only 34 out of 90 OPCRIT items are required to obtain a coherent DSM-IV diagnosis and that we could complete the items automatically using an algorithm based on the ODIP form.

All the process was first tested with 212 patients to avoid any computer generated errors. Then we compared results for all patients together and discordance between ODIP and OPCRIT diagnosis was then analysed to determine which corresponded better to the Clinician’s diagnosis when available (unavailable for 17 patients with discordant diagnoses).

**Results:**

88.2% of diagnoses for the 364 patients were equivalent when comparing ODIP and OPCRIT results. For the discordant diagnoses most of them (7.2%) were so mainly because of lack of needed information and when one of the systems provided a wrong diagnosis it was more often the OPCRIT (4.1%) than ODIP (0.5%).

**Discussion:**

We demonstrated the ability of our 13 item ODIP tool to provide more reliable diagnosis than OPCRIT in the context of first episode psychosis with no organic or toxic origin.

**Limitations:**

This tool was not intended to assess affective disorder diagnosis but only specify the diagnosis for the episode. As yet it was tested only on first psychotic episodes.

The primary interest of this new tool is the speed of administration and the relatively simple algorithm implemented in an excel file and available from the authors on request.

